# Les stries angioïdes

**DOI:** 10.11604/pamj.2014.17.13.3609

**Published:** 2014-01-14

**Authors:** Hakima Elouarradi, Karmane Abdelouahed

**Affiliations:** 1 Université Mohammed V Souissi, Service d'Ophtalmologie A de l'hôpital des spécialités, Centre Hospitalier Universitaire, Rabat, Maroc

**Keywords:** Stries angioïdes, syndrome maculaire, fond d'oeil, angioid streaks, macular syndrome, fundus

## Image en medicine

Patient âgé de 40 ans, se présente pour un syndrome maculaire (Baisse progressive de l'acuité visuelle, métamorphopsie) unilatéral de l'oeil gauche. L'examen clinique objective une acuité visuelle normale à 9/10e P2 de l'oeil droit, et diminuée à 3/10e P8 au niveau de l'oeil gauche. L'examen du fond d'oeil trouve un aspect des stries angioïdes au niveau des 2 yeux, avec une hémorragie maculaire au niveau de l'oeil gauche (A). L'angiographie à la fluorescéine ayant objectivé les stries angioïdes des 2 yeux avec des drusens colloïdes, associés à gauche à une hémorragie maculaire (B, C). La tomographie par cohérence optique (OCT) maculaire révèle des néovaisseaux choroïdiens associés à un décollement séreux rétinien hémorragique en grande partie rétrofovéolaire au niveau de l'oeil gauche (D). L'examen général est normal. Patient ayant bénéficié d'injections intravitréennes répétées de 1,25 mg de Bevacizumab (0,05CC) au niveau de l'oeil gauche avec une nette amélioration clinique (AV 7/10 P3 OG) et angiographique (E) dès la première injection. Patient toujours suivi en consultation avec prescription d'une auto surveillance régulière à la grille d'Amsler. Les stries angioïdes sont des ruptures de la membrane de Bruch, visibles sous la forme de lignes radiaires, sombres ou rougeâtres, partant de la papille. Habituellement asymptomatiques, ces stries peuvent se néovasculariser et entraîner un syndrome maculaire avec baisse marquée de l'acuité visuelle. Des associations à des pathologies générales sont classiquement décrites, dont le pseudoxanthome élastique principalement avec le risque de complications cardiovasculaires. Et ils peuvent être isolés comme le cas de notre patient. Les néovaisseaux choroïdiens auparavant responsables de malvoyance de manière inéluctable quelque temps après leur survenue sont désormais pris en charge par des injections intravitréennes de molécules anti-VEGF avec une efficacité certaine.

**Figure 1:**
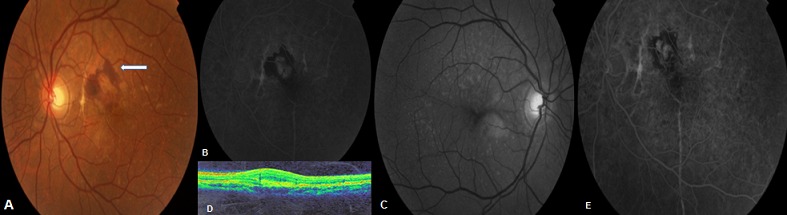
A) Fond d’œil OG: Stries rougeâtres, en rayon de roue péripapillaire avec hémorragie maculaire (Flèche) en périphérie d'un néo-vaisseau; B) Angiographie à la fluorescéine de l’œil gauche avant traitement; C) Angiographie à la fluorescéine de l’œil droit: aspect de stries angioïdes sans hémorragie maculaire; D) OCT de l’œil gauche: néovaisseaux choroïdiens; E) Angiographie à la fluorescéine de l’œil gauche après injection: nette diminution de l'atteinte maculaire.

